# Till Death Do Us Part: The Marriage of Autophagy and Apoptosis

**DOI:** 10.1155/2018/4701275

**Published:** 2018-05-08

**Authors:** Katrina F. Cooper

**Affiliations:** Department of Molecular Biology, Graduate School of Biological Sciences, Rowan University, Stratford, NJ 08084, USA

## Abstract

Autophagy is a widely conserved catabolic process that is necessary for maintaining cellular homeostasis under normal physiological conditions and driving the cell to switch back to this status quo under times of starvation, hypoxia, and oxidative stress. The potential similarities and differences between basal autophagy and stimulus-induced autophagy are still largely unknown. Both act by clearing aberrant or unnecessary cytoplasmic material, such as misfolded proteins, supernumerary and defective organelles. The relationship between reactive oxygen species (ROS) and autophagy is complex. Cellular ROS is predominantly derived from mitochondria. Autophagy is triggered by this event, and by clearing the defective organelles effectively, it lowers cellular ROS thereby restoring cellular homeostasis. However, if cellular homeostasis cannot be reached, the cells can switch back and choose a regulated cell death response. Intriguingly, the autophagic and cell death machines both respond to the same stresses and share key regulatory proteins, suggesting that the pathways are intricately connected. Here, the intersection between autophagy and apoptosis is discussed with a particular focus on the role ROS plays.

## 1. Introduction

Autophagy was discovered in 1963 as a lysosome-mediated degradation process for nonessential or damaged cellular constituents [1]. Since then, work pioneered in yeast [2, 3] has revealed that this widely conserved catabolic process is both highly regulated and a crucial integration point in cell physiology, [4, 5]. There are three main autophagic pathways that have been shown to coexist in mammalian cells called macroautophagy, microautophagy, and chaperone-mediated autophagy (CMA). Macroautophagy involves the formation of a doubled membrane structure called the autophagosome that fuses with the lysosome thereby transferring its luminal content for degradation [6]. Microautophagy refers to the process where cytosolic proteins are directly engulfed by the lysosome [7]. CMA, as its name suggests, utilzes cytosolic chaperones to deliver proteins to the surface of the lysosomes whereupon they unfold and cross the lysosomal membrane [8].

**Figure 1:**
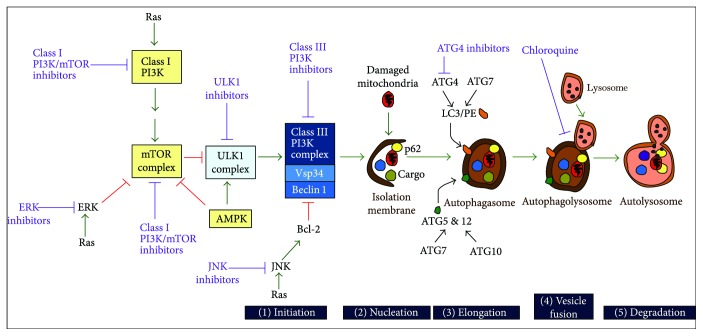
Schematic overview the five stages of the autophagy pathway. The execution point of where known pharmacological inhibitors act are written in purple. See text for details.

The subject of this review is the highly conserved process of macroautophagy, which here on out will be referred to as “autophagy.” Although more nuanced in higher eukaryotes, many of the AuTophaGy (Atg) genes and processes (outlined in Figure 1) initially defined in yeast are conserved [9, 10]. This significant body of work has also resulted in many different types of selective autophagy being identified. For example, mitophagy, pexophagy, and lipophagy represent the lysosomal degradation of mitochondria, peroxisome, and lipids, respectively. Given this wide range of substrates, understanding the molecular details of how the various components are both recognized and processed is now at the forefront of autophagy research [9]. Unfortunately, the recent explosion of published studies has also led to considerable terminology confusion. For example, the term canonical and noncanonical autophagy has been widely used in the literature to describe autophagy events that use different molecular signatures [11–13]. Recently, leaders in the field have reached a consensus on what these signatures should be called [10].

**Figure 2:**
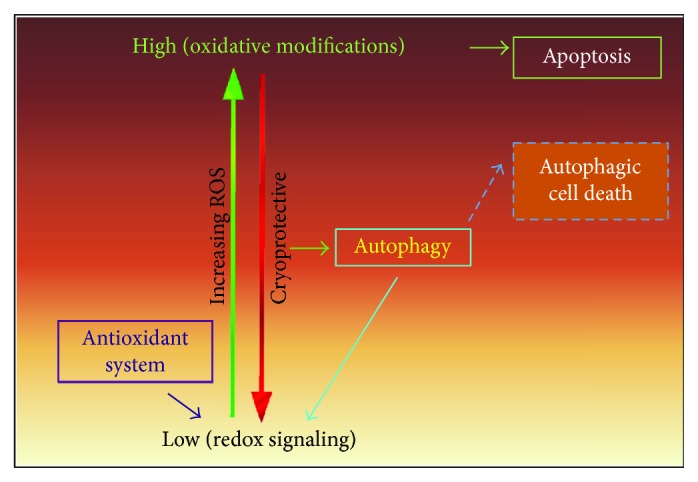
Diagram showing the closely linked relationship between ROS levels, autophagy, and apoptosis. See text for details.

As stated above, autophagy maintains cellular homeostasis under normal physiological conditions and in response to exogenous stimuli. Increased levels of intracellular reactive oxygen species (ROS) which arise predominantly from defective mitochondria also trigger autophagy. In turn, the increased autophagic flux drives down ROS by the consumption of damaged organelles (Figure 2). Thus, excess ROS upregulates autophagic flux, and in turn, this catabolic cellular process restores physiological ROS levels. As such, stimulus-induced autophagy underlies and sustains an adaptive response to stress with cytoprotective functions. However, when the levels of ROS become overwhelming, a nonautophagic regulated cell death (RCD) response is initiated suggesting that autophagy and RCD pathways are tightly linked [14]. How this switch is made is presently unclear. In this review, the relationship between ROS, autophagy and cell death are discussed. In addition, current knowledge about the crosstalk between autophagy and apoptosis is also reviewed. Lastly, cell death pathways have also been through a recent nomenclature classification [15]. For the purposes of this review, the type of RCD pathway will be referred to by its subtype. Thus, when referring to apoptosis, unless specified differently, I will be referring to both intrinsic and extrinsic mechanisms.

## 2. The Process of Autophagy

The word autophagy is fittingly derived from the Greek words for *self* (auto) and *eating* (phagy). It is a multistep catabolic process acting as a critical cellular response to nutrient and oxygen deprivation. Thereafter, free amino acids, free fatty acids, and ATP are recycled back into the cytoplasm for biomolecule synthesis. In mammals, there are five key control points, namely, initiation, nucleation, elongation, and lysosomal fusion and degradation of autophagosome contents. These stages are outlined in Figure 1, and the reader is referred to many excellent and recent reviews that provide more details on the role individual proteins play [16–18]. In short, initiation of the preautophagosomal membranes, which can be derived from the endoplasmic reticulum (ER) [19], begins with the activation of the ULK1 kinase complex. This complex is activated by cellular stress via mTORC inhibition and/or AMP-activated protein kinase (AMPK) activation [20]. ULK1 phosphorylates Atg13 and Fip200 to form an ULK1/2-mAtg13-Fip200 complex that is stabilized by Atg10 [16]. ULK1 activation promotes the recruitment of a multiprotein complex with class III phosphatidylinositol 3-kinase (PI3K) activity. This complex consists of 4 proteins which are scaffolded by Beclin-1, whose role upon release from the antiapoptotic protein Bcl-2, is to activate the vacuolar sorting protein Vps34 [21]. Maturation of the growing autophagosome membrane requires the complexes to recruit two ubiquitin-like conjugation systems. Both these systems involve the E1-like Atg7 [22, 23] which initiates the conjugation of LC3 with phosphatidylethanolamine (LC3/PE) and Atg5 with Atg12. Incorporation of these complexes into the autophagosome membrane is an essential process. Likewise, Atg4, the protease that cleaves and thereby activates LC3, is required the formation of the LC3-PE complex [24]. To end the program, the autophagosome fuses with the lysosome to form the autophagolysosome. The SNARE protein Stx17 [25] is essential for this process. Upon completion, the contents of the autophagosomes are degraded by the lysosomal hydrolases producing amino acids and lipids for protein and other macromolecular synthesis and metabolism.

In the past decade, the molecular mechanisms by which cargos are identified and consequently sequestrated within autophagosomes have been revealed. Best understood is the role the adaptor protein p62 plays in mitophagy. Here, p62 binds to defective and surplus mitochondria that are marked by ubiquitin thereby entrapping them in the autophagasome by binding to the autophagosome marker protein LC3 [26–28]. More recently, other selective autophagy receptors which include Nbr1, Ndp52, Vcp, and Optineurin have been characterized and the reader is referred to recent reviews for more detailed information [29, 30].

## 3. ROS Balance

How cells decide to switch from cellular homeostasis to apoptotic pathways upon ROS stress is not well understood. Understanding this is critical, as many types of cancers, especially established tumors, have adopted enhanced autophagy as a mechanism to survive in unfavorable environments. As a result, autophagic inhibition represents a new therapeutic tool to drive cells into regulated cell death (RCD) pathways [31]. However, a caveat to this approach is that, although rare, in some contexts, components of the autophagy machinery are used in autophagic cell death pathways [15, 17, 32]. Autophagic cell death is another area that has recently been redefined, the details of which are beyond the scope of this current review [10]. That being said, this reclassification is important as many reported studies of autophagic cell death may be due to defective apoptotic machinery [33]. However, autophagic cell death, although rare, does exist and is classified as an event that has to be retarded by pharmacological or genetic inhibition of autophagy. Given the fact that multiple components of the macroautophagy machinery have autophagy-independent functions ([34] and see below), it is recommended that before etiologically attributing a cell death event to macroautophagy, the involvement of at least two different proteins of the macroautophagy apparatus is shown to be required [10]. To date, three types of autophagic cell death have met with these more stringent criteria, autosis, ferroptosis, and more recently necroptosis [17, 35, 36]. Paradoxically, the loss of autophagy also contributes to de novo tumor formation, as autophagy is required to remove genotoxic materials that prevent malignant transformations [15]. Consistent with this hypothesis, mouse models of oncogene-driven caners with defective autophagy display accelerated tumor development. However, the tumors were benign and autophagy was essential for the progression to a more malignant state [33]. The favored model from these studies is that autophagy inhibits the initiation of tumorigenesis but promotes the survival of established tumors [37]. More recently, however, in certain contexts, the presence or absence of p53 and key Atg proteins dictates tumor growth in certain K-Ras-driven mouse models [38]. Thus, the relationship between autophagy, tumor suppressor genes, and oncogenes certainly warrants future studies.

### 3.1. ROS

**Figure 3:**
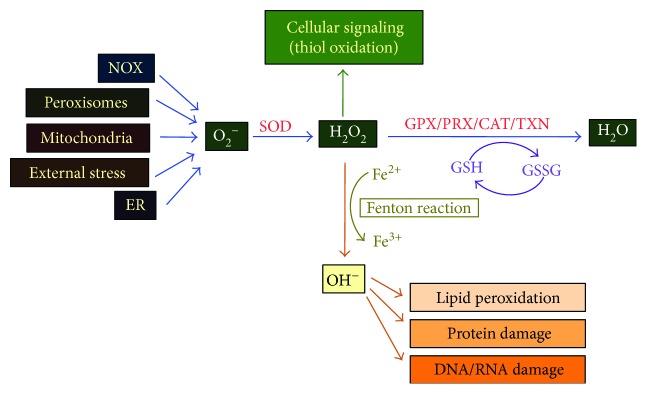
Schematic illustration of the mechanism involved in reactive oxygen species (ROS) formation and elimination. Endogenous forms of ROS arise from NADPH oxidase (NOX) as well as the organelles shown. The cytosolic superoxide (O_2_^−^) is converted into hydrogen peroxide (H_2_O_2_) by superoxide dismutase (SOD). H_2_O_2_ has three fates. It can be detoxified to water by glutathione peroxidase (GPX) peroxiredoxin (PRx), thioredoxin (TRX), and catalase (CAT). The reduced form of glutathione (GSH) promotes this reaction whereas oxidation to glutathione disulfide GSSH results in intracellular redox. H_2_O_2_ can be converted to the cytotoxic hydroxyl radical (OH^−^) via the Fenton reaction resulting in irreversible damage to lipids, proteins, and DNA. Lastly, H_2_O_2_ can also be used as a signaling molecule by oxidizing critical thiols within proteins to regulate numerous biological processes.

ROS is classified as a heterogeneous group of molecules generated naturally in cellular metabolism from diatomic oxygen [39]. The group includes the highly reactive free oxygen radicals (superoxide anion O_2_^−^, hydroxyl radical OH^−^) and the stable ‘diffusible' non-radical oxidant, hydrogen peroxide (H_2_O_2_). Their formation begins with the univalent reduction of oxygen to produce superoxide radical O_2_^−^ (see Figure 3). This predominantly occurs in the mitochondria as a result of electron leakage during normal respiration in the electron transport chain [40]. O_2_^−^ is also produced from other sources: in peroxisomes through *β*-oxidation of fatty acids and flavin oxidase activity [41]; in the endoplasmic reticulum (ER) from protein oxidation of molecular oxygen [42]; and by enzymatic reduction of molecular oxygen with xanthine/xanthine oxidase, uncoupled nitric oxide synthases (NOS), cytochrome P-450 isoforms, and NADPH-dependent oxidases (NOXs) being key contributors [39]. As O_2_^−^ is highly reactive with the ability to convert to the toxic OH^−^ radical, it is rapidly converted to the more stable and membrane-diffusible ROS H_2_O_2_ [43]. This occurs either spontaneously or through the actions of superoxide dismutases (SOD1 and SOD 2 [44]).

### 3.2. Antioxidants

How cells process intracellular H_2_O_2_ is intricately linked to their cell fate. It can be converted to water (ROS detoxification) or the genotoxic hydroxyl radical by different enzymes. Lastly, it can be used as a signaling molecule in a process coined redox signaling (Figure 3). ROS detoxification is executed by a variety of enzymes, the key players being catalase, glutathione peroxidases (GPXs), peroxiredoxins, glutathione peroxidases (GSH-Px or GPx), and thioredoxin (TXN). Whilst PRXs are associated with H_2_O_2_ scavenging, the GPX family of proteins (GPX1–8) catalyzes the reduction of H_2_O_2_ to H_2_O by oxidizing reduced glutathione (GSH) to glutathione disulfide (GSSG). Consistent with this, GSH oxidation to GSSG results in intracellular redox imbalance which is reflected by a decreased GSH:GSSG ratio [45]. Other antioxidants are vitamin C, vitamin E, and carotenoids. Conversion of H_2_O_2_ to the damaging free hydroxyl radicals occurs by the Fenton reaction where free iron (Fe^2+^) reacts with H_2_O_2_. This insoluble radical has strong oxidizing potential and causes irreversible oxidative damage to virtually any cellular macromolecules within the vicinity of their production [46, 47]. Thus, cellular levels of H_2_O_2_ and OH^−^ are maintained by a balance between oxidant and antioxidant responses.

### 3.3. Role of Transcription Factors

The transcription factor Nrf2 (nuclear factor erythroid 2-related factor 2) [48] plays a key role in both ROS detoxification, prevention of OH^−^ production, and redox balance [49]. Following exposure to oxidants or electrophiles, Nrf2 accumulates in the nucleus where it upregulates four groups of genes encoding detoxification and antioxidant enzymes. These include those needed for the biosynthesis and maintenance of GSH [50], cytosolic thioredoxin (TXN), thioredoxin reductase (TXNRD), and sulfiredoxin (SRXN), all of which reduce oxidized protein thiols [51]. In addition, genomic studies have revealed that Nrf2 regulates over 600 genes [52] including those required for inhibition of inflammation and the repair or removal of damaged proteins. This has resulted in Nrf2 being named “the master regulator of antioxidant responses” [53]. As befitting this principal role, Nrf2 itself is tightly controlled by ubiquitin-mediated proteolysis which is inhibited following oxidative stress (see below for details) [54].

Worth mentioning is that Nrf2 indirectly helps to modulate ROS levels by regulating free Fe(II) homeostasis. This is achieved by the upregulation of genes encoding members of the ferritin complex, which detoxifies Fe(II) by converting it into Fe(III). This complex also sequesters iron within its own structure that prevents it from being accessed by the Fenton reaction, thus reducing the production of OH^−^ radicals from ROS [55, 56]. Given this role, it comes as no surprise that iron excess can significantly promote tumorigenesis [57, 58]. This has led to the emergence of using iron chelation or transferrin receptor-neutralizing antibodies, to treat cancer [59]. However, the molecular mechanisms by which iron excess promotes tumorigenesis remain unclear. Recently, the tumor suppressor p53 was identified as a protein that can ligate the heme iron using one cysteine side chain. This promotes p53 nuclear export and degradation by ubiquitin-mediated proteolysis [60]. Lastly, although beyond the scope of this review, it is important to mention that other transcription factor families, for example, Forkhead box O (FoxO) and nuclear factor-*κ*B (NF-*κ*B), also regulate antioxidant gene expression [53].

## 4. ROS as a Signaling Molecule

Since the 1990's, the model that cellular oxidant production is inherently damaging has been replaced by a more complex scenario in which regulated oxidant production functions as important physiological regulators of intracellular signaling pathways [61]. These include cellular proliferation and differentiation as well as stress-responsive programs [62]. This posttranslational modification is achieved by H_2_O_2_-mediated oxidation of reactive cysteine residues found within redox-sensitive signaling proteins. Importantly, this reaction, in which the sulfhydryl group undergoes deprotonation and oxidation, is reversible being easily reduced back to reduced cysteine by either enzymatic systems (thioredoxin/thioredoxin reductase system) or nonenzymatic reactions (thiol/disulfide exchange). This reversibility provides the on/off switch, a character of that is essential for signaling. An emerging theme is that antioxidant proteins also actively participate in redox signaling [61]. For example, they catalyze the reduction of oxidized proteins as well as binding to signaling intermediates thereby activating downstream effectors such as p38 MAPK and the c-Jun N-terminal kinase (JNK) [61]. However, when ROS levels cannot reach homeostasis, the reversible SOH derivative can be hyperoxidized to the irreversible and damaging SO_2_H derivative [63].

Given the potential of this posttranslational modification to affect a wide range of cellular processes, large-scale proteomic approaches have been used to identify proteins that potentially possess modulatory cysteine residues [64–66]. The results identified many phosphatases that are well established signaling molecules [67, 68]. A more recent study has identified that many mitochondrial proteins contain potentially reactive cysteines [69]. Intriguingly, apart from the protease Atg4, no other autophagy proteins were identified in these screens. However, two groups have proposed that the superoxide acts as a signal to activate mitophagy by depolarizing the mitochondrial inner membrane. These depolarized mitochondria become fragmented and recruit Park2, the mitophagy E3 ubiquitin ligase [70, 71].

## 5. RNS as a Signaling Molecule

Although not the subject of this review, in addition to ROS, cells contain reactive nitrogen species (RNS) mostly in the form of nitric oxide (NO^−^). Nitric oxide is generated by the mitochondria and acts as a cell signaling molecule in many physiological processes including mitochondrial biogenesis and bioenergetics [72, 73]. NO^−^ itself is not highly toxic as it is efficiently removed by its rapid diffusion through tissues into red blood cells, where it is converted to nitrate by reaction with oxyhemoglobin [74]. However, when both superoxide O_2_^−^ and NO^−^ are synthesized within a few cell diameters of each other, they will combine spontaneously to form peroxynitrite (ONOO^−^) that can mediate cellular damage in a wide range of conditions [75]. Small amounts of peroxynitrite may also spontaneously decompose to yield NO_2_^−^ and the hydroxyl radical [76]. Similar to ROS, RNS can add posttranslational modifications to proteins by S-nitrosylation of reactive cysteines [77]. Importantly, Drp1, the GTPase that regulates mitochondrial fission, is posttranslationally modified in such a manner [78]. In addition, several proteins that bind to the core fission/fusion proteins also contain redox-sensitive motifs [79]. Taken together, this data suggests that RNS and ROS both regulate mitochondrial morphology via posttranslational modification.

## 6. Direct Effect of ROS on Autophagy

**Figure 4:**
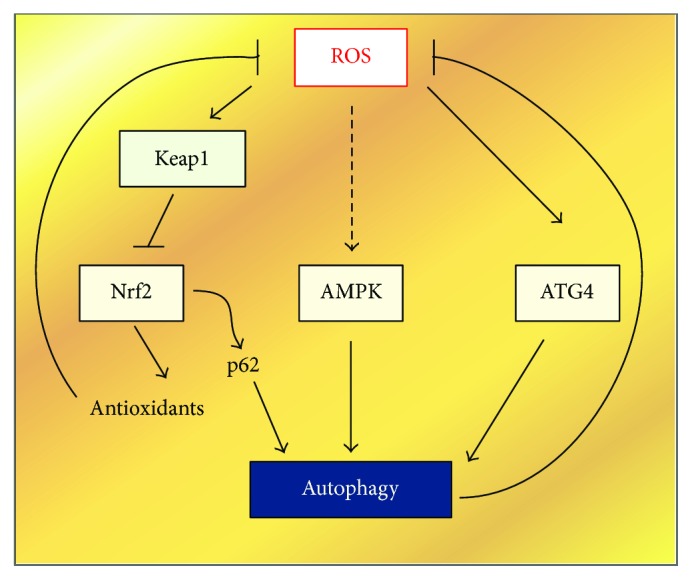
Diagram depicting the genetic relationship between ROS and autophagy initiation. The dotted lines represent an indirect relationship.

It is well established that ROS can induce autophagy, as this is a major mechanism used to exsanguinate superfluous cellular ROS. In turn, autophagy drives down the levels of ROS as it consumes damaged mitochondria, the major source of ROS. This “pas de deux” represents a finely tuned negative feedback mechanism by which autophagy eliminates the source of oxidative stress and protects the cell from oxidative damage. Increased intracellular ROS that is accompanied with increased autophagic flux is triggered by many factors including starvation, hypoxia, TNF*α* (tumor necrosis factor *α*), and NGF (nerve growth factor) deprivation [80, 81]. Consistent with this, studies have shown that treatment of make cells with the ROS scavenger N-acetyl cysteine (NAC) decreases both cellular ROS production and autophagy, implicating redox thiol signaling as an important regulator of autophagy. Likewise, exogenous H_2_O_2_ and suppressed NF-*κ*B activation of mTOR mimics these effects [82]. These findings are consistent with H_2_O_2_ effects being mediated through the production of ROS and redox signaling. However, the precise molecular details on how ROS crosstalks with the autophagic machinery are still unclear. In the last few years, it has emerged that redox imbalance has a pivotal role in driving the process. Consistent with this, two proteins Atg4 and Keap1, which have opposing roles in promoting or inhibiting autophagy, respectively, are regulated by redox signaling. Lastly, AMPK, which is a major inducer of autophagy in response to starvation, may also indirectly play a role (see Figure 4).

### 6.1. ATG4

Atg4 is the only mammalian protein whose redox regulation has been shown to be necessary for the progression of autophagy [24]. Atg4 is a protease which is active in a reducing environment where it cleaves the C-terminal domain of LC3. This allows LC3 conjugation with phosphoethanolamine (PE) which is a hallmark of, and necessary for, autophagosome formation. Upon oxidation of an active cysteine residue (C81), the protease activity is inhibited, resulting in increased autophagy. These results were obtained in 2007, and although it has also been speculated that other enzymes involved in the initiation and elongation stages of autophagosome formation may also be regulated by redox signaling, no concrete evidence has been reported.

### 6.2. AMPK

AMPK an established indirect regulator of autophagy [20, 83, 84]. During normal physiological conditions, cellular homeostasis is maintained by strictly matching the generation and consumption of ATP. When ATP levels become low, they are replenished by autophagic recycling of unnecessary cytoplasmic material, such as misfolded proteins, supernumerary, or defective organelles [18]. AMPK is critical for this process as it is an energy sensor, being activated by increased levels of ADP and AMP [84, 85]. This AMP:ATP imbalance can be stimulated by multiple stresses including amino acid starvation, glucose withdrawal, hypoxia, and H_2_O_2_ [86]. Given this role, it is not surprising that AMPK is regulated by the intracellular redox status, being activated by Trx1 during energy starvation which promotes access by AMPK to two key cysteine residues in the catalytic subunit [87]. Once activated, AMPK can initiate autophagy by several ways. It negatively regulates components of the mTOR signaling cascade [88, 89] as well as directly activating the ULK1 kinase [90] (Figure 1). Furthermore, in yeast, it has recently been shown that following glucose starvation, the AMPK homologue Snf1 is recruited to the outer mitochondrial membrane, where it phosphorylates the Atr homologue Mec1. This is then required for recruitment of Atg1 (ULK1 homologue) thereby allowing the Snf1-Mec1-Atg1 module to maintain mitochondrial respiration by initialing autophagy during glucose starvation [91]. Although the molecular mechanisms still remain unclear, Atg1 may maintain mitochondrial respiration by directly or indirectly phosphorylating key mitochondrial proteins which are essential for respiration [91].

The very fast induction of autophagy following ROS exposure suggests that a rapid on/off molecular switch may regulate initiation of autophagy. Some research has implicated that AMPK could play a role as, following hypoxia, it is activated in an AMP:ATP-independent manner [92, 93]. In support of a rapid switch, is the observation that following ROS induction, GSH is excluded from cells. This consequently permits the accumulation of redox-sensitive proteins in their oxidized form. Also, chemically oxidized GSH can induce autophagy in the absence of an autophagic stimulus [94, 95]. This result serves to strengthen the key role redox homeostasis plays in autophagy commitment.

### 6.3. Others

Other proteins also indirectly respond to increased cellular ROS. These include high mobility group box 1 (Hmgb1, a nuclear protein that is released extracellularly in response to cytokines), Ras, and various kinases, Atm, Akt, Erk, JNK, and Perk to name a few [96]. In recent years, the role these proteins play in regulating autophagy has become increasingly important as their signaling capabilities have been linked to cancer cell progression [97]. A classic example is oncogenic Kras signaling, which is an established driver of pancreatic ductal adenocarcinoma (PDAC). More recently, tumor growth has been shown to be contingent on stromal inputs that are derived from fibroblasts of the pancreatic tumor microenvironment [98]. These observations have initiated test therapies that couple an established autophagy inhibitor (chloroquine) with kinase inhibitors [33].

### 6.4. Keap1 and p62

Different to Atg4, the Kelch-like ECH-associated protein 1 (Keap1) is a redox-sensitive protein that indirectly negatively regulates autophagy in response to ROS [99]. Keap1 serves as a substrate adaptor protein for the (Keap1)-Cullin 3 (Cul3) E3 ubiquitin ligase complex [100]. Under normal physiological conditions, this complex is responsible for the rapid turnover of the transcription factor Nrf2. However, Keap1 is equipped with reactive cysteine residues which, upon exposure to oxidants, causes a conformational change which impairs its ability to trap Nrf2 for ubiquitylation and degradation [101]. The resulting stable Nrf2 then translocates to the nucleus where it upregulates antioxidant genes [102]. Stable Nrf2 can also be created by competitive binding of p62 to the Nrf2-binding site on Keap1. p62 as mentioned above, is the autophagic adaptor protein that brings dysfunctional mitochondria to the phagosome [103]. Therefore, increased free p62 levels activate the Nrf2 pathway. p62 is also an Nrf2 target gene, thus creating a positive regulatory loop [104]. p62 also promotes the expression of other signaling proteins including NF-*κ*B and mTor1 [105, 106] and thus has gained notoriety as a signaling hub [107]. Intriguingly none of these functions depend on the ubiquitin-associated or LC3-interacting region domains of p62 [105], but they are linked to cytosolic p62 levels which are regulated by autophagy via by its LIR domain that binds to LC3 on autophagasomal membranes [105]. As in vivo studies have shown that overexpression of p62 is carcinogenic in hepatocellular carcinoma [108], it has been suggested that homeostatic maintenance cytosolic p62 levels contributes to the final outcome of the tumorigenic process [107]. This has led to the idea that a critical role of autophagy is to prevent p62-driven tumor initiation and malignant transformation.

## 7. Autophagy and Apoptosis—Till Death Do We Part

**Figure 5:**
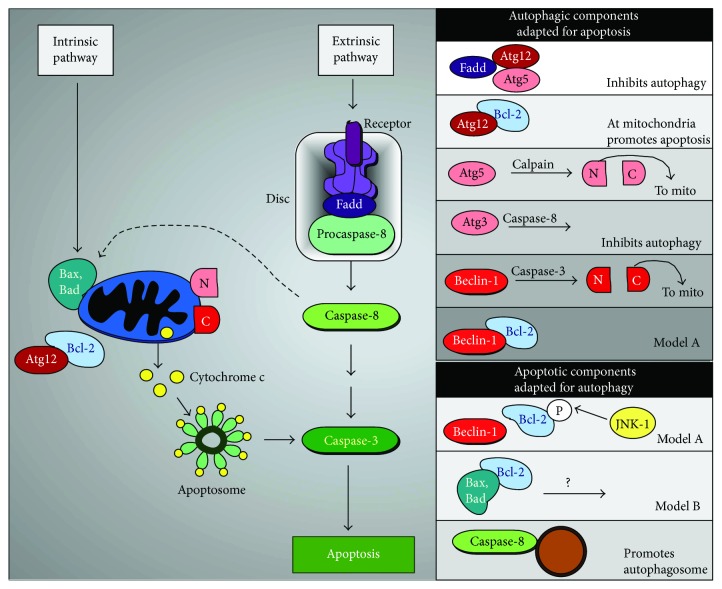
Diagram showing the intricate relationship between autophagy and the extrinsic and intrinsic apoptotic pathways. See text for details.

Both autophagy and apoptosis respond to similar stresses. However, the molecular mechanisms that dictate cell fate decisions are only just emerging. What is striking is that proteins that were originally thought to be required for just one pathway have now been shown to play a role in both. Thus, the decision to commit cellular suicide following stress may be controlled by many factors as opposed to a simple molecular switch. What is also apparent is that our understanding of how apoptosis repurposes the ATG machinery to promote cell death far outweighs the current knowledge of how autophagy inhibits apoptosis. This is somewhat surprising as there are a large number of examples in the literature where autophagy protects against apoptosis. The salient points of this symbiotic relationship are discussed below and summarized in Figure 5. Further details can be found in many excellent reviews and original papers cited therein [14, 109–112]. The overwhelming recent explosion of data has though served to emphasize that, like all marriages, the relationship is complex. What is becoming clear however is that in a narcissistic manner, each pathway steals and adapts proteins from the other pathway to promote its own mechanism.

### 7.1. Brief Outline of Apoptosis

The cast of characters that play a role in the intersection of apoptosis and autophagy are derived from two distinct but connected apoptotic pathways, intrinsic and extrinsic apoptosis (outlined in Figure 5) and described in many excellent reviews [113, 114], so only the salient details are given below. The *intrinsic pathway* is characterized by pro- and antideath signals converging at mitochondrial membranes. These consequently become permeabilized (MOMP—mitochondrial outer membrane permeabilization), leading to the release of mitochondrial intermembrane proteins including cytochrome c. Rapid cell death follows as MOMP triggers both caspase activation on the apoptosome and blocks caspase inhibitors. Together, this starts a cascade of active caspases which cleave hundreds of cellular substrates ending in cellular demise. The Bcl-2 family of proteins consists of proapoptotic and prosurvival proteins which together control MOMP. Under basal conditions, the prosurvival proteins, Bcl-2, Bcl-X_L_, and Mcl-1, inhibit MOMP in two ways. First, they directly bind and inhibit the proapoptotic effector proteins Bax and Bak, which form the pores in the mitochondrial membrane. Second, they bind to BH3-only proteins such as Bim which prevents them from activating Bax [115].

The *extrinsic* receptor-mediated apoptosis pathway is triggered by the ligation of death receptors with their cognate ligands. This stimulates receptor clustering resulting in the recruitment of cytoplasmic adapter proteins, important amongst which is Fadd. Fadd then associates with procaspase-8 leading to the formation of a death-inducing signaling complex (DISC). This results in the dimerization and catalytic activation of caspase-8, which can then directly cleave and activate caspase-3 [116]. Both intrinsic and extrinsic pathways result in caspase-3 activation that is linked to the initiation of the execution phase of apoptosis. Crosstalk between the two pathways is mediated by caspase-8 cleavage and activation of BID. BID is a BH3-interacting domain death agonist, the product of which (truncated BID; tBID) is required in some cell types for death receptor-induced apoptosis [117].

### 7.2. Beclin-1 and Bcl-2

The best described relationship between autophagic and apoptotic proteins is the complex relationship between Beclin-1, the antiapoptotic proteins, Bcl-2 [118] (plus family members Mcl1-1 and Bcl-X_L_) and the prodeath protein Bax [119]. In this ménage à trois Bcl-2 plays a key role as under normal physiological conditions, its interaction with Beclin-1 and Bax inhibits autophagy and apoptosis, respectively [118]. Currently, there is no consensus on the molecular mechanisms that define this relationship. Furthermore, it is complicated as there are two distinct cellular pools of Bcl-2, one at the ER where it is bound to Beclin-1, [118, 120], and the other at mitochondria where it is bound to Bax. In the original model, (model A, in Figure 5) under autophagy-inducing conditions, a BH3-only protein, (either Bik, Bad, or Nova) competitively binds to Bcl-2, thereby displacing it from Beclin-1 [118, 121]. This displacement is augmented by JNK1 phosphorylation of Bcl-2 and required for Beclin-1 to activate Vps34 resulting in the nucleation of an isolation membrane thereby promoting autophagy [122]. As Bcl-2 has a higher affinity for Bax than Beclin-1, this low level phosphorylation of Bcl-2 is not enough for it to be dissociated from mitochondrial Bax. This proapoptosis move occurs if the stress signal becomes overwhelming and requires Bcl-2 hyperphosphorylation [122].

Many inducers of autophagy also cause cell death, which lead David Vauz and colleagues to challenge this model. In a series of elegant genetic and biochemical experiments, his group demonstrated that in the absence of Bax and Bak, antagonizing or altering the levels of prosurvival Bcl-2 family members has no detectable impact on autophagy [121]. This then suggests a model (model B in Figure 5) in which the effects of Bcl-2 on autophagy are an indirect consequence of its inhibition of apoptosis by associating with Bax and Bad. Thus, as both Beclin-1 and Bcl-2 are key regulators of autophagy and apoptosis, respectively, it is imperative that these opposing models be resolved. As it seems to be the case in many studies, both models could be correct but context specific.

### 7.3. Caspases

Caspases are cysteine proteases that traditionally are principle mediators of apoptotic cell death [123]. In recent years, they have been shown to shift the balance of cellular homeostasis towards apoptosis by dismantling several key Atg proteins, including Atg3, Vps34, and Beclin-1. The culprit caspases are 3 and 8 that cleave PI3K members (Vps34 and Beclin-1) and Atg3, respectively [124–126]. Worthy of note is that the proteolytic product of Beclin-1 (and caplain cleaved Atg5, see below) translocates to the outer mitochondrial membrane and exhibits a proapoptotic activity [125, 127]. Thus, the apoptotic machinery not only inactivates autophagy but also repurposes proteins to promote cell death. Consistent with this theme, the cleavage of Beclin-1 is enhanced by Bax thereby further suppressing autophagy [127]. As neither the N- nor C-terminal fragments of Beclin-1 can interact with Vps34, the cleavage of Beclin-1 has been shown to be a critical event whereby caspases inhibit autophagy [128, 129]. Consistent with this, a noncleavable Beclin-1 mutant can restore autophagy [130].

Unexpectedly, it has also been shown that caspases can also promote autophagy under certain contexts. As stated above, caspase-8 is activated by DISC, a multiprotein signaling platform. In the absence of DISC, caspase-8 can still be activated from procaspase-8 by being recruited to autophagosomes. Its localization to the autophagosome this is executed by binding either to the autophagic cargo receptor p62 or through an interaction between the adaptor protein Fadd and Atg5 [128]. It remains unclear if this mechanism promotes apoptosis or autophagy pathways as both scenarios have been reported in the literature in different tumor types [128, 131]. The most likely scenario is that these contradictory functions are likely to be context specific. Ascribing which function caspase-8 is playing at the autophagosome is worth while as this mechanism has been successfully exploited to render cancerous cell lines responsive to further pharmacological treatment [132].

Other caspases also have been reported to have proautophagic roles [133]. Similar to caspase-8, their prosurvival survival role at present appears to be context specific [134–136]. For more details, I refer the reader to an excellent recent review [133]. Further research needs to be executed to define the exact mechanism by which these caspases execute their proautophagic role. To summarize the relationship of caspases with apoptosis is surprisingly complex, with different caspases augmenting prosurvival or death pathways [133].

### 7.4. Atg5 and Atg12

Atg5 and Atg12 are two members of the ubiquitin-like conjugation systems that are needed for autophagosome formation. As stated earlier, the ubiquitin-like protein Atg12 is transferred from Atg7 the E1-like enzyme, via Atg10 (E2-like) to form a covalent attachment with Atg5 [137–139]. The Atg12–5 conjugate is essential for autophagy. More recently, procell death roles have emerged for both Atg5 and Atg12 in their unconjugated forms. Atg5 is cleaved by caplains (which are cysteine proteases activated by cellular stress) and plays a key role in the initiation of apoptosis [140]. Following cleavage, the N-terminal of Atg5 translocates to the mitochondria, where it mediates the release of cytochrome c by interacting with Bcl-X_L_ to promote apoptosis [141]. More recently, unconjugated Atg12 also been ascribed a procell death function by two quite separate autophagy-independent mechanisms. Firstly, free Atg12 binds to and inactivates mitochondrial Bcl-2 family members [142]. Secondly, Atg12 can conjugate to Atg3 where it promotes mitochondrial fusion and restricts mitochondrial mass [143]. Lastly, although not involved in mediating apoptosis, the Atg12–Atg3 conjugate promotes basal autophagy and endolysosomal trafficking [144]. Taken together, these observations serve to demonstrate the flexibility of repurposing Atg proteins for different roles dependent upon the cellular circumstances. Despite these discoveries, it remains to be seen if any or a combination of these interactions represents a key event where autophagy and apoptosis diverge in response to specific signals.

## 8. Conclusions

This review has summarized how cells maintain cellular ROS levels as well as discussing the complicated relationship between autophagy and apoptosis. The last decade has seen an explosion of reports that has lead to an increased understanding of how these pathways communicate. Importantly, the overriding theme evolving from these studies is that the relationship between autophagy and apoptosis is intertwined, with proteins from both pathways being repurposed for the benefit of the other. What is also emerging is that these new roles (also known as *night job*) of proteins whose “*day job*” is firmly established are, in many cases, context specific. Given the consideration that many chemotherapeutic regimes both inhibit apoptosis and/or autophagy [5, 33, 97], it is of great importance that both the molecular mechanisms and context-specific job assignment be defined for these multifunctional proteins.
